# Mitigating the Accumulation of Mercury (Hg) and Lead (Pb) through Humic Acid Application under Aquaponic Conditions Using Watercress (*Nasturtium officinale* R. Br.) as a Model Plant

**DOI:** 10.3390/plants13172386

**Published:** 2024-08-27

**Authors:** Judit Éva Lelesz, József Csajbók, Péter István Molnár, István Csaba Virág, Erika Tünde Kutasy

**Affiliations:** 1 Department of Animal Husbandry, Institute of Animal Science, Biotechnology and Nature Conservation, Faculty of Agricultural and Food Sciences and Environmental Management, University of Debrecen, Böszörményi Str. 138, H-4032 Debrecen, Hungary; lelesz.judit@agr.unideb.hu (J.É.L.); molnar.peter.istvan@agr.unideb.hu (P.I.M.); 2 Department of Crop Production, Applied Ecology and Plant Breeding, Institute of Crop Sciences, Faculty of Agricultural and Food Sciences and Environmental Management, University of Debrecen, Böszörményi Str. 138, H-4032 Debrecen, Hungary; csj@agr.unideb.hu (J.C.); virag.istvan.csaba@agr.unideb.hu (I.C.V.)

**Keywords:** heavy metal bioaccumulation, lead (Pb), mercury (Hg), humic acid, element content, aquaponics

## Abstract

In aquaponic farming, there is a potential risk that heavy metals will contaminate the water, which can lead to heavy metal accumulation in the plants. Our research investigated the accumulation of mercury (Hg) and lead (Pb) under aquaponic conditions and the effect of their increased presence on the uptake of other macro- and micronutrients using watercress (*Nasturtium officinale*) as a model plant. The potential modifying effect of humic acid on heavy metal accumulation was also investigated. Adding Hg and Pb increased the mercury and lead levels of the watercress plants to over 300 µg kg^−1^, while the addition of humic acid significantly reduced the concentration of both mercury and lead in the plants compared to plants treated with heavy metals alone, from 310.647 µg kg^−1^ to 196.320 µg kg^−1^ for Hg and from 313.962 µg kg^−1^ to 203.508 µg kg^−1^ for Pb. For Fe and Mn, higher values were obtained for the Hg + humic acid treatments (188.13 mg kg^−1^ and 6423.92 µg kg^−1^, respectively) and for the Pb + humic acid treatments (198.26 mg kg^−1^ and 6454.31 µg kg^−1^, respectively). Conversely, the Na, K, Cu levels were lower compared to those in plants treated with heavy metals alone. Our results demonstrated that watercress can accumulate mercury, leading to high levels, even above food safety standards, highlighting the importance of water quality control in aquaponic systems. Furthermore, these results suggest that watercress could be used as a natural filter in recirculation systems. The addition of humic acid significantly reduced the accumulation of heavy metals and altered the element content in the plant.

## 1. Introduction

The world population is growing, which is having an impact on the environment. Increasing food production will require the development of efficient technologies. Due to climate change and desertification, countries are facing droughts with scarce water resources (drought-prone regions) and we need innovative agricultural practices to ensure food security and food safety. Aquaponics is a sustainable food production system combining hydroponics (soil-less crop production) and aquaculture (fish farming) [1]. The establishment of aquaponics systems has two general objectives. The first is to utilise the organic matter content of the run-off water from intensive systems. The second is to reduce the environmental load of the substances present in this run-off water. This environmental load may result from the high levels of nitrogen substances in this type of run-off, which can increase eutrophication in natural waters. One objective could be to reduce these nitrogen levels while simultaneously generating economic profit. Recirculating aquaculture systems recycle effluent from fish to reduce pollution, and aquaponics further reduces the organic matter content in the water used. This production system can be integrated into the circular economy, increasing the efficiency of inputs for food production. The hydroponic subsystem of an aquaponics system cleans water of organic contaminants, which then can be reused for fish farming. By combining the two subsystems, fish and crop farming becomes much more environmentally friendly. It also has the advantage of producing eight times as much food per unit area while using less water than conventional agriculture [2].

Due to the economic and environmental advantages of aquaponics, the market is predicted to expand by 2030, and it is anticipated to reach USD 2.3 billion, with a compound annual growth rate (CAGR) of 13.7% over the 2023–2030 period [3]. Currently, the North American region dominates the global aquaponics market [4]. The use of aquaponics is widespread in the European research segment, but more technological innovation and investment will be needed to develop a profitable industry [5]. The European aquaponics market is projected to grow at a CAGR of 13.2% over the next period (2023–2030), with Germany leading the market and expected to do so in the future, with the market estimated to reach a value of USD 140.1 million by 2030. The UK (12.2% CAGR) and French (14% CAGR) markets are also developing rapidly [2].

The most commonly grown crops in aquaponics systems are fruits (pineapples, blueberries, strawberries and mandarins), herbs and leafy vegetables (e.g., pak choi, water spinach, lettuce, parsley, basil, mint and watercress) [3,6,7,8]. Watercress (*Nasturtium officinale*) is a member of the *Brassicaceae* family and an aquatic plant used for human consumption and medicinal treatments [9]. It has a pungent and peppery taste and can be eaten raw or cooked. This low-calorie, low-fat plant is rich in vitamins (vitamins C, B and K) and minerals (iron, iodine, calcium and phosphorus) and contains significant amounts of amino acids and antioxidants. The demand for watercress is emerging in urban areas and exceeds the available supply [10,11], thus its cultivation is expected to expand in the future. Watercress is consumed fresh in ready-to-eat salad mixes and has been used as food and medicine for over 2000 years [12]. Some studies have shown that the consumption of vegetables, including watercress, has chemopreventive effects by protecting against lung cancer [13]. The consumption of watercress may reduce basal DNA damage [14]. Watercress is also likely to be well-suited for phytoremediation, as it can accumulate nickel and store most of it in its roots [15]. Researchers found that watercress can efficiently accumulate macro- and microelements, which affect the plant contents of glucosinate, phenolic acid and antioxidants, among other substances [16].

Ecosystems are contaminated with heavy metals through human activities, which is why heavy metal pollution has become a focus of environmental safety [17,18,19]. Even small amounts of heavy metals can be quite hazardous and can accumulate in living organisms [20]. The bioaccumulation of chemicals in biota can have adverse effects on the ecosystem [21]. Plants grown in urban environments can reduce pollution through bioaccumulation. Heavy metals are found in the tissues of these plants, mainly from transport emissions in urban areas, but also from mining, manufacturing, fertiliser production, pesticide use and water pollution [22,23].

The decline in animal populations in Europe is partly due to the continuing pollution of the aquatic environment [24]. Persistent and bioaccumulative toxic substances ingested by fish pose a health risk. These can be lipophilic contaminants (artificial) and monomethyl mercury (naturally occurring). Omnivores can introduce mercury and organohalogens into their milk, meat and eggs [25]. Humans are exposed to heavy metals through food, soil, air, water and the products that they use [26]. Heavy metals can enter the human body through ingestion, inhalation and direct contact [27]. The body stores them in fatty and bone tissues and they can cause a number of diseases if mobilised [28]. Since certain salts of these elements are highly soluble in water, they move relatively easily in water [22].

Lead and mercury are dangerous heavy metals that can easily enter aquaponics systems, accumulate in the plants grown there and cause health problems if ingested. Mercury is known as an ultra-trace element, as it has no known function in the human body. Consequently, there is no regulation of this element, so it accumulates easily. Its sources include dentistry, fungicides, electrical equipment and paint manufacturing. The two main sources of mercury are dental amalgam and fish (methylmercury). Mercury exposure in humans is mainly caused by food. Most of the mercury accumulates in fish in its organic form. Minamata disease is caused by eating shellfish and fish contaminated with mercury. It is therefore necessary to monitor the fish intended for human consumption. Many animal feeds contain fishmeal and if precautions are not taken, farm animals can also become contaminated with mercury [29]. Fish can accumulate mercury in large quantities and are therefore considered the largest source of mercury in human diets [30]. The amount accumulated depends on the trophic level, age and length of the fish. For example, predatory fish have higher mercury concentrations than other fish species [31]. Some research has indicated that certain fish species should be avoided by pregnant women because of the high risk to the foetus from mercury contamination [32]. Before 2000, tetraethyl lead was added to petrol to prevent engine “knock”. Although now banned, it has contributed to lead pollution [33]. According to research by Zhang et al. [34], hydrophytes from sediments can more readily accumulate Cd, Cu, Pb and Ni.

There is some evidence for enhanced plant tolerance to the uptake of heavy metals by using humic acid. Humic acid added in appropriate amounts can improve the phytoremediation of sediments contaminated with heavy metals, by increasing the accumulation of heavy metals in plants grown in contaminated soil, such as *Mentha aquatica* L, *Typha orientalis* Presl and *Tagetes patula* L. [34]. In field observations, humus products were found to enhance the response of plants to drought stress and to give plants greater resistance to saline soil conditions. However, in the low-stress or no-stress settings, humic acid had a much smaller effect than expected on the yield of the crops [35,36]. Humic acid supplementation above a certain level can have the opposite effect. This humic acid threshold depends on the plant species and type of growing medium [37]. Tattini et al. [38] reported that the nitrogen uptake rate of the roots of olive plants increased after application of humic acid, but with greater humic acid concentrations decreasing nitrogen uptake. Valdrigbi et al. [39] also found that humic acids enhance mineral nutrient uptake by plants by increasing the permeability of root cell membranes, but this phenomenon varies from species to species.

In an aquaponics system, fish, plants and microorganisms live in close contact with each other. Fish feed is the nutrient source for the aquaponics system, providing the necessary nutrients for the fish, plants and microorganisms. The environmental load of heavy metals in water can come from lead components in old systems and from fish feed. The main source of dietary protein in fish feed is fishmeal made from marine fish [40,41,42,43,44,45]. About two thirds of the world’s fishmeal production is used for aquafeeds [46]. Heavy metals are a major pollutant in the aquatic environment and can accumulate in marine fish and easily enter the aquaponic system with the feed. If this heavy metal contamination in natural waters and soils is more common in the area, aquaponics could use its economic profit-making role as a remediation filter to prevent the release of these toxic heavy metals into natural waters and soils.

The aim of our study was to investigate the effect of heavy metal (mercury and lead) contamination in aquaponics water on the Hg and Pb content of the plant, in this case, watercress, and the effect of heavy metal accumulation on the element content of the plant. Furthermore, our objective was to investigate how the presence of humic acid in aquaponics systems affects the uptake of heavy metals, such as lead and mercury, as well as the metabolism of other nutrients in relation to these substances in the plant.

## 2. Results

### 2.1. Effect of Mercury and Humic Acid Treatment on the Elemental Content of Watercress

**Figure 1:**
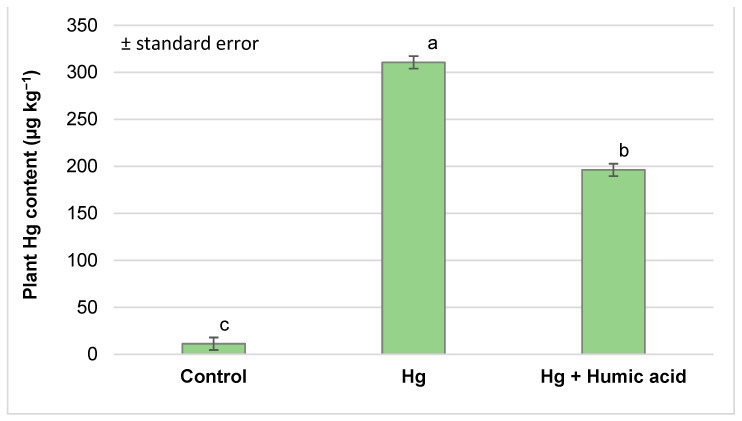
Mercury accumulation in watercress plants grown in mercury-contaminated water and the effect of humic acid on Hg accumulation (Debrecen, 2023); mean ± SE of three replicates. Different letters indicate statistically different values (*p* < 5%).

Measurements taken on day nine of the experiment showed that the mercury content of the plant samples from the different treatment groups differed significantly. The treated watercress plants were able to accumulate a high amount of mercury (310.65 µg kg^−1^) compared to the control group, which was clearly and significantly reduced by the addition of humic acid to the system (196.32 µg kg^−1^), as shown in Figure 1. Despite the beneficial effect of humic acid, the mercury content of the plants was significantly higher than that of the untreated control plants (11.28 µg kg^−1^).

**Figure 2:**
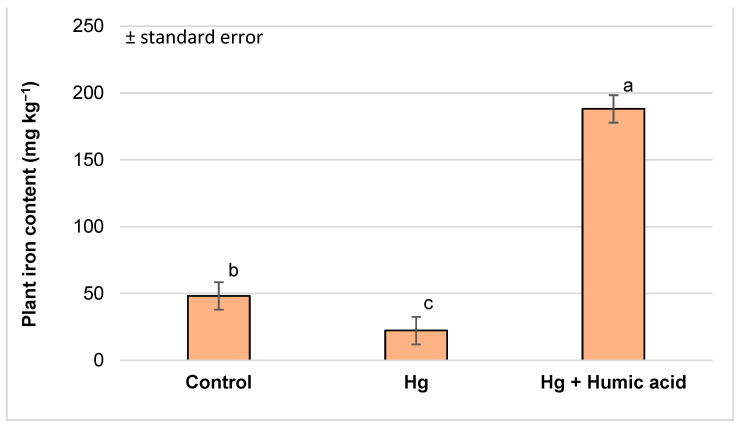
Iron content in watercress plants grown in mercury-contaminated water and the modifying effect of humic acid on Fe accumulation (Debrecen, 2023); mean ± SE of three replicates. Different letters indicate statistically different values (*p* < 5%).

The iron content of the plant samples was significantly different between the control (48.21 mg kg^−1^), mercury (22.29 mg kg^−1^) and mercury + humic acid (188.13 mg kg^−1^) treatment groups. The mercury-treated group showed a decrease, whereas when humic acid was added to the system, the plants had a significantly higher iron level compared to the control group. Thus, the excessive presence of mercury decreased the iron content of the plant, while the application of humic acid not only compensated but also greatly increased it (Figure 2).

**Figure 3:**
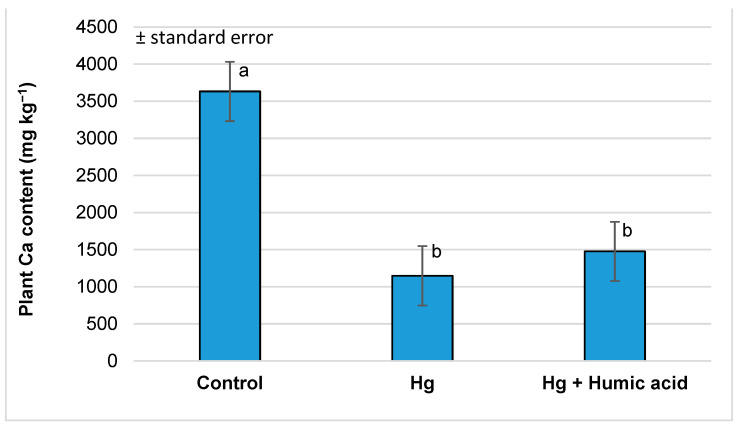
Calcium content in watercress plants grown in mercury-contaminated water and the modifying effect of humic acid on Ca accumulation (Debrecen, 2023); mean ± SE of three replicates. Different letters indicate statistically different values (*p* < 5%).

**Figure 4:**
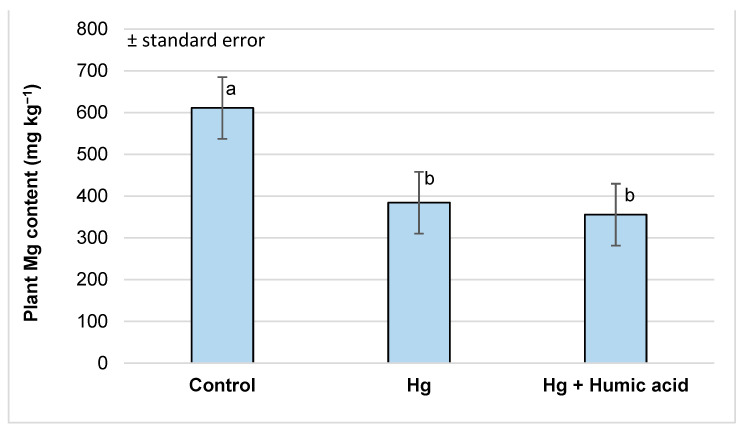
Magnesium content in watercress plants grown in mercury-contaminated water and the modifying effect of humic acid on Mg accumulation (Debrecen, 2023), mean ± SE of three replicates. Different letters indicate statistically different values (*p* < 5%).

Examining the calcium and magnesium content of the watercress samples, we found that the mercury treatment significantly reduced the amount of these elements in the plant shoots (Ca: from 3632.86 to 1148.93 mg kg^−1^; Mg: 610.91 to 384.25 mg kg^−1^). This was not significantly affected by the addition of humic acid (Figure 3 and Figure 4).

In Table 1, it can be observed that the sodium content did not change significantly with the Hg and Hg + humic acid treatments, although a decrease was observed, and the situation was similar for copper. For potassium, the differences were significant and humic acid increased the amount of this element in the watercress compared to the Hg treatment. This effect was even more pronounced for zinc and manganese. For manganese, this difference exceeded 50%, while the values for zinc were 260% higher than those of the control group.

### 2.2. Effect of Lead and Humic Acid Treatment on the Elemental Content of Watercress

**Figure 5:**
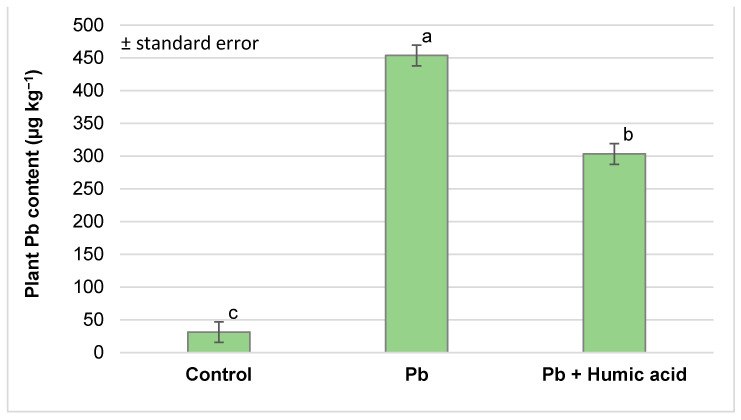
Lead accumulation in watercress plants grown in lead-contaminated water and the effect of humic acid on Pb accumulation (Debrecen, 2023); mean ± SE of three replicates. Different letters indicate statistically different values (*p* < 5%).

When measuring the lead content of the plant samples, we obtained results quite similar to those for mercury. The lead treatment increased the lead content of the watercress in the system by several times (453.75 µg kg^−1^) compared to the control (31.21 µg kg^−1^). With the addition of humic acid, the lead content of the plants was statistically significantly reduced compared to those treated with lead alone, although it was still significantly higher (303.34 µg kg^−1^) compared to the control plants (Figure 5).

**Figure 6:**
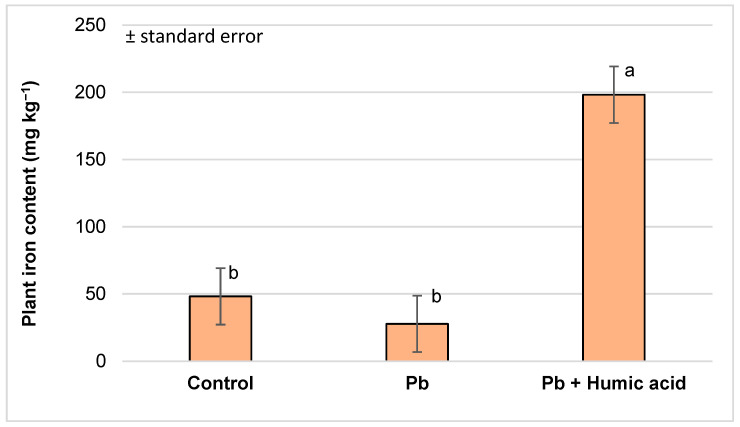
Iron content in watercress plants grown in lead-contaminated water and the modifying effect of humic acid on Fe accumulation (Debrecen, 2023); mean ± SE of three replicates. Different letters indicate statistically different values (*p* < 5%).

By analysing the iron content of the plants in the lead-treated plants, the following conclusions can be drawn. The iron content of the plant samples decreased with the lead treatment, but this was not significant. Similar to the mercury treatment, the iron content of the samples was significantly higher in the lead + humic acid-treated plants due to the effect of the humic acid. The increase was 411% compared to the control and 715% compared to the plants treated with lead alone (Figure 6).

**Figure 7:**
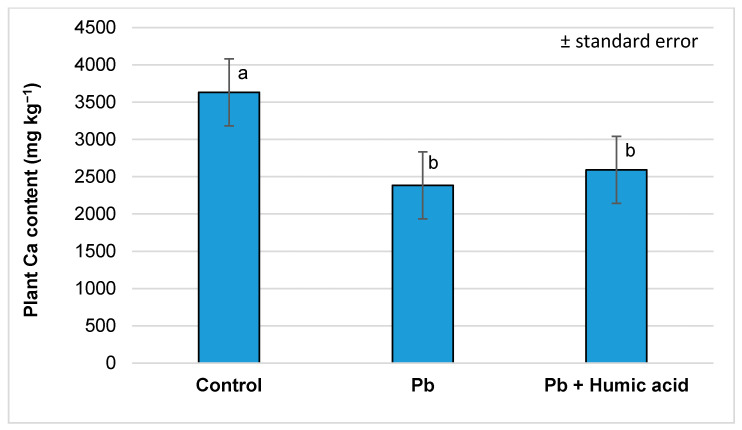
Calcium content in watercress plants grown in lead-contaminated water and the modifying effect of humic acid on Ca accumulation (Debrecen, 2023); mean ± SE of three replicates. Different letters indicate statistically different values (*p* < 5%).

**Figure 8:**
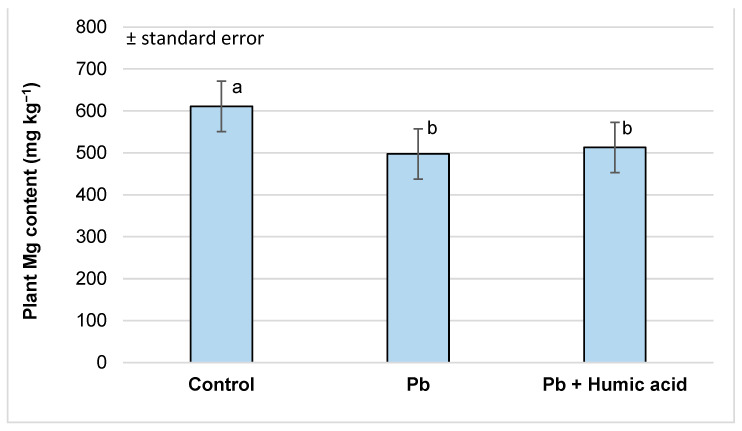
Magnesium content in watercress plants grown in lead-contaminated water and the modifying effect of humic acid on Mg accumulation (Debrecen, 2023); mean ± SE of three replicates. Different letters indicate statistically different values (*p* < 5%).

The lead treatment caused a significant decrease in calcium and magnesium levels (-65.6% and -81.4%, respectively) compared to the control group. As with mercury, the humic acid treatment did not cause a significant difference compared to the lead-only group (Figure 7 and Figure 8).

The lead treatment resulted in a significant decrease (32.3%) in the sodium content compared to the control group, which was not significantly affected by the addition of humic acid. For potassium, no significant difference between the treatments was found. When measuring the manganese content, a similar phenomenon was observed for the Pb + humic acid treatment and the Hg + humic acid treatment: a significant increase of almost 50%. This phenomenon was not observed for zinc, and no verifiable difference between the treatments was found. The lead treatment reduced the copper content by 12.9% compared to the control, although the reduction was not significant. The addition of humic acid caused a further reduction, which was significant compared to both the control and lead-only groups. The copper content of the plants was 58.7% of the value measured in the control group (Table 2).

### 2.3. Effect of Mercury, Lead and Humic Acid Treatments on the Water Quality 

**Figure 9:**
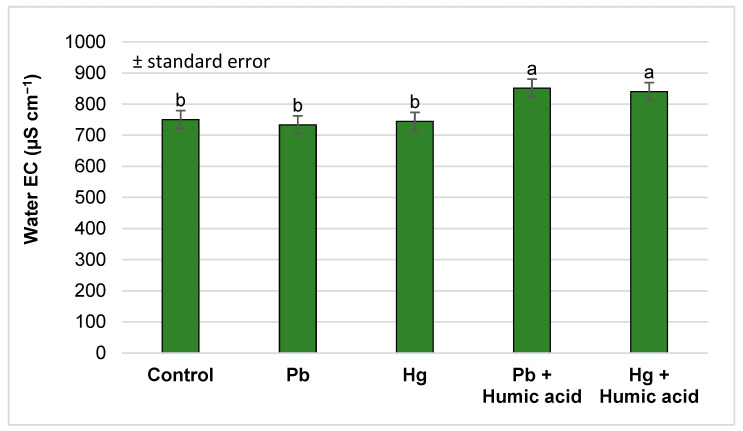
Changes in water electrical conductivity under the different treatments (Debrecen, 2023); mean ± SE of three replicates. Different letters indicate statistically different values (*p* < 5%).

The mercury and lead treatments did not cause any measurable change in the EC values of the water compared to the control. However, significant increases were observed for both the lead and mercury groups with the addition of humic acid (Figure 9).

**Figure 10:**
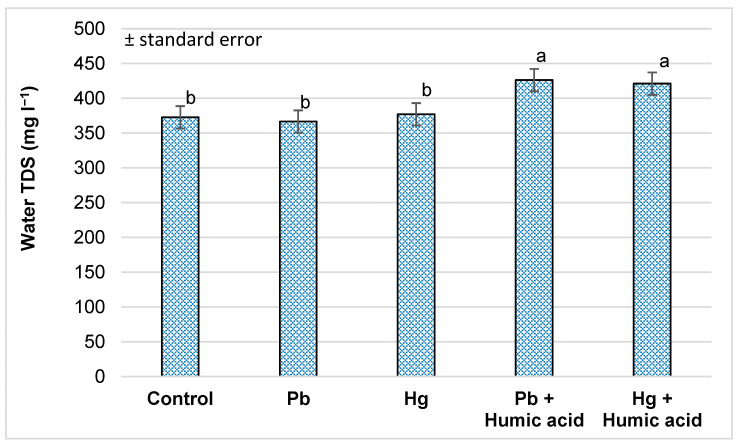
Changes in water TDS under different treatments (Debrecen, 2023); mean ± SE of three replicates. Different letters indicate statistically different values (*p* < 5%).

The amount of total dissolved solids (TDS) in the water did not change in the lead and mercury treatment groups compared to the control group. In contrast, there was a significant increase in the Pb + humic acid (116%) treatment and Hg + humic acid (112%) treatment groups compared to the heavy metal-only treatment group (Figure 10).

**Figure 11:**
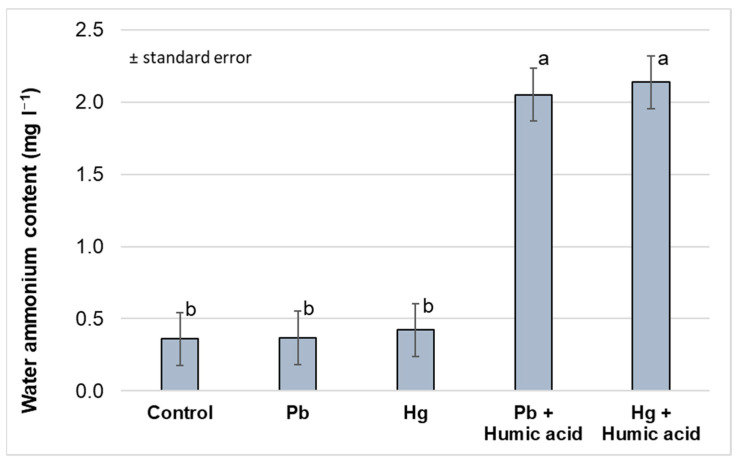
Changes in water ammonium content under different treatments (Debrecen, 2023); mean ± SE of three replicates. Different letters indicate statistically different values (*p* < 5%).

**Figure 12:**
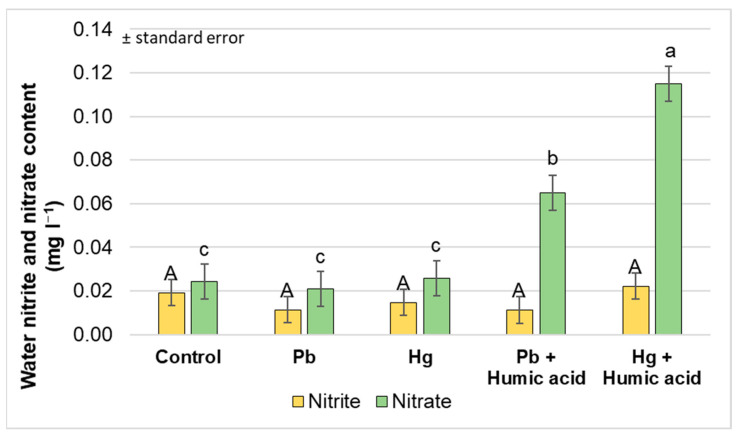
Changes in water nitrate and nitrite contents under different treatments (Debrecen, 2023); mean ± SE of three replicates. Different letters indicate statistically different values (*p* < 5%).

The nitrogen forms in the aquaponic water showed no significant difference between the heavy metal-only (mercury and lead) treatment groups compared to the control group. There did not appear to be a significant difference between the control group and the heavy metal treatment groups. In contrast, significant increases in ammonium and nitrate levels were observed under the treatments to which humic acid was added. The nitrite levels were low in the system. The high nitrate levels in the Hg + humic acid treatment group indicate that the bacterial flora was functioning and converting ammonium and nitrite to nitrate. The bacterial activity was apparently also functioning in the control group, but not as efficiently as in the humic acid-treated group (Figure 11 and Figure 12). The addition of humic acid to both the lead and mercury treatments significantly increased the levels of ammonium nitrogen (459.5% and 406.3%, respectively) compared to the heavy metal-only treatments.

As with ammonium, the nitrate levels increased significantly in the humic acid treatment groups compared to the control group and the groups treated with heavy metals alone. This suggests that humic acid also increases the amount and rate of ammonium transformation in the aquaponic system, although this is not supported by the unchanged nitrite levels that were measured in the system.

## 3. Discussion

The high organic matter content of run-off water from intensive fish farming systems is an untapped resource. Aquaponics systems based on these waters, which are no longer needed for fish farming and often cause eutrophic pollution, would allow for the secondary use of these waters and the production of crops in addition to primary aquaculture products. However, in aquaculture, especially in intensive fish farming systems, the presence of heavy metals, such as mercury and lead, can have a significant polluting effect. The impact of potential heavy metal pollution on watercress (*N. officinale)* model plants was tested, as previous research proved that several species in the *Brassicaceae* family can accumulate various heavy metals [47,48].

In our study, using a 50 mg kg^−1^ Hg treatment in the form of a Hg(NO_3_)_2_ solution, nine days after the heavy metal treatment, the watercress accumulated large amounts of mercury (310.65 µg kg^−1^, Figure 1). Taking into account that mercury can be very dangerous even at low concentrations due to its high toxicity and bioaccumulative capacity, our result is considered to be a very high value. According to Hungary’s Joint Decree No. 17 of 1999 [49], the permitted Hg content of dried vegetables should not exceed 50 µg kg^−1^ dry matter. The European Food Safety Authority (EFSA) established Provisional Tolerable Weekly Intakes (PTWIs) of 4 μg/kg bw (body weight)/week for total mercury and 1.3 μg/kg bw/week for methylmercury [50].

Several studies [51,52,53,54,55,56,57,58,59] have investigated the accumulation capacity of heavy metals (Cd, Cr, Cu, Ni, Zn, Co and As) in watercress, but no research data on mercury were found. Sinulingga et al. [60] investigated the phytoremediation capacity of water spinach (*Ipomoea aquatica*) and found that using a concentration significantly lower than our concentration (maximum 5 mg kg^−1^ Hg treatment in the form of a Hg(NO_3_)_2_ solution), the Hg content of water spinach increased to 116 µg kg^−1^ after 15 days of treatment. The lower concentration of Hg in the treatment and the shorter duration did not cause Hg accumulation in the plant. In our experiment, the lead treatment (50 mg kg^−1^ Pb(NO_3_)_2_ solution) resulted in a lead content of 453.75 µg kg^−1^ in watercress (DAT 9) (Figure 5), which, like the mercury content in our experiment, exceeds the 300 µg kg^−1^ allowed in the European Union [61]. Khan et al. [62] investigated the bioaccumulation of Pb and other heavy metals (Cd, Zn and Cu) in wild watercress that grew along the Swat River Basin (Pakistan) and found that it was very high: 50–115 mg kg^−1^ concentration of Pb in the edible parts of watercress plants that grew in areas with a high ecological risk index. The consumption of these plants raises health concerns. In Saudi Arabia, Al Jassir et al. [63] measured the lead content of leafy vegetables, including watercress, sold by the roadside and found dangerously high levels of Pb (660 µg kg^−1^ in washed plants and 840 µg kg^−1^ in unwashed plants), which were significantly higher than the levels of accumulated Pb from the lead treatment in our experiment. Kumar et al. [50] recorded high lead accumulation in some other *Brassica* species too. The Pb content in the roots ranged from 0.82 to 10.9% of the dry weight. In particular, *Brassica juncea* (L.) showed a high accumulation capacity (108.3 mg g^−1^ Pb in the roots and 34.5 mg g^−1^ in the shoots).

The mineral content of watercress is exceptional in itself, as confirmed by the study of Martín-León et al. [64]. They examined 56 samples of watercress (*Nasturtium officinale*) in their study. Among the leafy vegetables tested, watercress showed outstanding values for iron (12.78 mg kg^−1^), zinc (3733 µg kg^−1^), copper (490.5 µg kg^−1^) and manganese (3491.7 µg kg^−1^). In our experiment, we measured an even more favourable element content (Fe: 48.21 mg kg^−1^; Zn: 5363.51 µg kg^−1^; Mn: 4209.09 µg kg^−1^), especially for Cu (3767 µg kg^−1^) (Figure 2, Table 2). In in vitro cultures of *N. officinale,* Klimek-Szczykutowicz et al. [16] found that it accumulated micronutrients more efficiently than macronutrients.

The copper content (13,790 µg kg^−1^ in washed and 27,020 µg kg^−1^ in unwashed plants) and Zn content (34,290 µg kg^−1^ in washed and 41,130 µg kg^−1^ in unwashed plants) in the watercress sold along the road in Saudi Arabia measured by Al Jassir et al. in 1995 [63] was significantly higher than our results. In our study, the Cu content of the watercress was not significantly affected by the Hg (2486.87 µg kg^−1^), Hg + humic acid (2747.92 µg kg^−1^), Pb (3281.22 µg kg^−1^) or Pb + humic acid (2211.38 µg kg^−1^) treatment. On the contrary, Broadley et al. [47], in their work on metal accumulation in angiosperms, found that the correlation between lead and copper uptake was moderate. However, they found no association between the Pb and Zn contents [47]. This is similar to our results, where neither the Pb (5609.34 µg kg^−1^) nor Pb + humic acid (5287.56 µg kg^−1^) treatment affected the Zn content of the watercress. However, it is interesting to note that although the Zn content of the plant did not change after the Hg treatment (4869.32 µg kg^−1^), when humic acid was added to the mercury, the Zn content increased significantly (13,984.50 µg kg^−1^), by more than 2.5 times (Table 1 and Table 2).

The results from Sahu et al. [65] found that the tissues of mercury-treated wheat plants showed a decrease in their K, Ca and Mg contents (0.201, 0.342 and 0.346 mmol g^−1^) compared to the control (0.391, 0.623 and 0.723 mmol g^−1^). Similarly, in our investigations, we found that in watercress, the levels of Ca and Mg were significantly lower in both the Hg (Ca: 1148.93 mg kg^−1^; Mg: 348.25 mg kg^−1^; Figure 3 and Figure 4) and Pb (Ca: 2348.18 mg kg^−1^; Mg: 497.42 mg kg^−1^; Figure 7 and Figure 8) metal treatment groups compared to the control group (Ca: 3632.86 mg kg^−1^; Mg: 610.91 mg kg^−1^) (Figure 3, Figure 4, Figure 7 and Figure 8). Regarding potassium, the mercury treatment also significantly decreased the K content (control: 1109.79 mg kg^−1^; Hg: 838.84 mg kg^−1^; Table 1); in contrast, the Pb treatment did not affect the K content (control: 1109.79 mg kg^−1^; Pb: 1129.36 mg kg^−1^; Table 2) of the plants.

Several plant processes are affected by humic substances, including enzyme activity, protein metabolism, photosynthesis, respiration, and water and nutrient uptake [66,67,68,69,70,71]. Studies have shown that humic acid is an excellent foliar fertilizer carrier and activator that increases the carbohydrate content, promotes higher yields or improves product quality, and primarily affects plant nutrition and growth by promoting the growth of the shoots and roots [72]. Baldotto and Baldotto [73] and Ampong et al. [74] found that humic acid can increase the efficiency of light, water, and nutrient use by promoting hormonal balance in plants, favouring plant metabolism.

The addition of humic acid to aquaponic systems can significantly reduce the amounts of mercury and lead that accumulate in watercress. In our study, the concentration of mercury (310.65 µg kg^−1^) and lead (453.75 µg kg^−1^) in the plant significantly decreased in the presence of humic acid (Hg + humic acid: 196.32 µg kg^−1^; Pb + humic acid: 303.34 µg kg^−1^), although these heavy metal amounts were still significantly higher than that of the control (Figure 1 and Figure 5). In their study, Liu et al. [75] found that the Hg contents in the rice plants in the control group were 0.72, 4.29 and 0.61 μg kg^−1^ (dry weight) in the straw, roots and grains, respectively. The addition of humic acid (HA) at three different Hg/HA concentration ratios (50, 25 and 10) decreased the Hg content by 35.28–69.86% in the different plant organs. Lower Hg/HA concentration ratios resulted in a stronger effect. They stated that humic substances can alleviate the growth suppression induced by heavy metals in diverse plant species cultivated under many different conditions. The mitigation of Hg accumulation by humic acid in plants is dependent on the dose of humic acid [75]. Moreno et al. [76] reported that mercury–thiosulphate complexes could be translocated and accumulated in the upper parts of the plants, reaching up to 25 times the Hg concentration in the substrate.

Zhang et al. [77] also found that the addition of humic acid could be an effective way to reduce the Pb concentration in tobacco leaves grown on polluted acidic soils. In tobacco plants grown in non-polluted soils, the Pb content of the leaves was 7.29–9.55 mg kg^−1^, while in plants grown in polluted (500 mg kg^−1^ Pb) soils, it was 13.77–37.23 mg kg^−1^. The application of humic acid reduced Pb accumulation in the tobacco leaves by 19.44% and 30.22% when the plants were grown in acidic red and paddy soils; on the contrary, in the alkaline cinnamon soil, the addition of humic acid increased the leaf Pb concentration by 11.69%. They stated that humic acid could be used to reduce metal accumulation in plants growing in polluted acidic soils and could be used for metal bioremediation in alkaline soils.

Iron deficiency is a very common problem in aquaponics systems; although there could be iron in the sludge of the system, it is in an insoluble form and is thus not available to the plants. Through chelation (tying insoluble ferric iron ions to organic chelating agents like humic acid), we can keep iron soluble and biologically available for plants [78]. In our experiment, compared to the control (48.21 mg kg^−1^), the plants’ uptake of iron strongly increased in the presence of humic acid (Hg + humic acid: 188.13 mg kg^−1^; Pb + humic acid: 198.26 mg kg^−1^; Figure 2 and Figure 6). Chen et al. [79], using a hydroponics system, proved that the addition of humic acid or fulvic acid enhances the Fe solubility and availability for both monocotyledonous (ryegrass) and dicotyledonous (soybean and melon) plants. Iron chelation [78] could be an effective way to reduce the occurrence of common iron deficiency diseases in aquaponics systems.

However, in our experiment, no significant changes in calcium and magnesium uptake were observed in the mercury + humic acid (Ca: 1476.06 mg kg^−1^; Mg: 355.52 mg kg^−1^) and lead + humic acid (Ca: 2591.59 mg kg^−1^; Mg: 512.83 mg kg^−1^) treatment groups compared to the mercury-treated (Ca: 1148.93 mg kg^−1^; Mg: 348.25 mg kg^−1^) and lead-treated (Ca: 2384.18 mg kg^−1^; Mg: 497.42 mg kg^−1^) plants (Figure 3, Figure 4, Figure 7 and Figure 8). In another study, Bulut and Akinci [80], in a similar vein to us, found that the application of 10 mL/L of HA from Leonardite plus 30 mL of Hoagland solution did not significantly increase the micronutrient and calcium uptake of broad bean plants.

In our experiment, humic acid did not mitigate the negative effect of the heavy metal treatments on the Na (Hg + humic acid: 475.56 mg kg^−1^; Pb + humic acid: 524.77 mg kg^−1^) and Cu (Hg + humic acid: 2747.92 µg kg^−1^; Pb + humic acid: 2211.38 µg kg^−1^) contents of watercress. In addition, the potassium levels in the plant samples from the mercury + humic acid treatment groups were similar to that of the control group but significantly different compared to the treatment groups without humic acid. The humic acid treatments considerably increased the Mn content of the watercress (Hg + humic acid 6423.92 µg kg^−1^; Pb + humic acid: 6454.31 µg kg^−1^) compared to the heavy metal treatments (Hg: 2657.73 µg kg^−1^; Pb: 3375.10 µg kg^−1^), even above the control (4209.09 µg kg^−1^). Sönmez et al. [81] observed that the application of humic acid had a significant effect on *Tagetes erecta* plants’ concentrations of N, P, K, Mg, Na, Fe, Zn, Cu, Mn, Cd, Ni and Pb. They found the lowest values for Fe, Zn and Cu (340.8, 13.9 and 0.79 mg kg^−1^, respectively) in the control plants. With humic acid application, the Zn and Cu contents decreased in the plants, while the Mn content increased substantially from 102.4 mg kg^−1^ to 156.7 mg kg^−1^. Foliar application of humic acids increased the uptake of P, K, Mg, Na, Cu and Zn of corn [82]

Exceeding a certain threshold can worsen this positive effect of humic acid treatments. It would be worthwhile to determine this threshold value in aquaponics systems, although this will also be influenced by the properties of the model plant.

The significantly higher ammonium and nitrate contents in the water in the heavy metal + humic acid treatments (Figure 12) suggest that the bacterial flora were more effective at counteracting the negative effects of heavy metals. This phenomenon and the quantitative changes in the concentrations of heavy metals and nutrients in the water were not the focus of our experiments, but we consider it worthwhile to extend our research in these directions in the future.

## 4. Materials and Methods

The experiments took place at the Fish Biology Laboratory, Faculty of Agricultural and Food Sciences and Environmental Management, University of Debrecen. The aim of the experiments was to investigate watercress’s (*Nasturtium officinale* R. Br.) ability to bind heavy metals under aquaponic culture conditions.

Experiment design

Fifteen small-scale, independent aquaponics systems without growing medium were set up in a double-walled, shaded greenhouse made of plastic tunnels. The water was not returned to the fish due to the use of heavy metals in the experiment. Each stand-alone system consisted of a 110 L plant tank and a 225 L water tank, with water flow provided by a Tetra Tec WP 1000 water pump for each system (Tetra GmbH, Melle, Germany). The 15 systems were completely independent of each other. The aquaponic systems were filled with water on day 0 of the experiment, with 150 L of water per system. The water and nutrient supply for the systems came from a fish rearing system with a net volume of 12 m^3^, which contained an average population density of 20–25 kg m^−3^ of a mixed stock of carp. The average pH of the water was 8.43.

At the start of the experiment, 100 watercress shoots with a length of 20 cm were placed in each system. No conventional growing medium was used, and only plastic netting was used to keep the plant shoots above the water surface.

The treatments were as follows:

1. Control (no heavy metals or humic acid were added to these systems);
2. Pb (50 mg kg^−1^) (25 mL of a 48% Pb(NO_3_)_2_ solution);
3. Pb (50 mg kg^−1^) (25 mL of a 48% Pb(NO_3_)_2_ solution) + H (humic acid) (1000 mg kg^−1^) (150 mL);
4. Hg (50 mg kg^−1^) (25 mL of a 0.01 mol l^−1^ Hg(NO_3_)_2_ solution);
5. Hg (50 mg kg^−1^) (25 mL of solution) + H (humic acid) (1000 mg kg^−1^) (150 mL).

The Pb(NO_3_)_2_ and the Hg(NO_3_)_2_ were obtained from Sigma-Aldrich, St. Louis, MO, USA. The humic acid was purchased from Humin Aqua AGRI-TECH BIO s.r.o. Brestovec, Slovakia. There were 3 replicates per treatment.

Measurements

On days 0, 1, 3 and 9 of the experiment, we made measurements and took plant and water samples. We recorded the oxygen saturation (%), dissolved oxygen level (mg L^−1^), water temperature (°C) (HACH HQ30d flexi), chemistry (pH) and amount of total dissolved solids (TDS). The pH measurements were made using a HANNA Combo pH and ORP waterproof pH tool and Adwa AD 332 (Adwa Hungary, Szeged, Hungary). For air temperature (°C) and relative air humidity (%) measurements, we used a PCE-THB 40 Humidity/Baro/Temp. Data Recorder (PCE Holding GmbH, Meschede, Germany).

We took 50 mL water samples from each system to measure the ammonium (NH_4_^−^N) (HACH Method 8038), nitrite (NO_2_^−^-N) (HACH Method 8507) and nitrate (NO_3_^−^-N) (HACH Method 8192) levels using a HACH Lange DR3900 spectrophotometer (HACH Company, Loveland, CO, USA).

For the laboratory measurements, three 10 cm pieces of plant samples per group were taken. All the 15 systems were sampled, with 3 replicates per treatment. The plant samples were stored frozen until the laboratory measurements were performed.

Element measurements

Hg and Pb: Excavation was performed under pressure according to the Hungarian standard MSZ EN 13805:2015 [83] using a microwave digestion system (MARS 6, CEM Corp. Matthews, NC, USA). The plant samples were first homogenised and then 1–2 g was weighed with 1 milligram accuracy and transferred into a digestion vessel. A 2.5 mL volume of concentrated nitric acid and 0.5 mL of concentrated hydrogen peroxide were pipetted onto the sample. After resting for a few hours, another dose of 2.5 mL of concentrated nitric acid and 0.5 mL of concentrated hydrogen peroxide was added to the sample. The samples were then diluted with distilled water to 50 mL.

The Hg content of the plant samples was determined according to the MSZ EN 13806:2002 standard [84]. The analysis was performed using a Thermo Fisher iCE 3300 AAS atomic absorption spectrophotometer (Thermo Fisher Scientific, Waltham, MA, USA) and the cold vapour technique. The digested sample was reduced with sodium borohydride and transferred to the spectrophotometer. The light absorption of the solution was measured at a wavelength of 257.3 nm, which is proportional to the concentration of mercury in the solution. The standard solution used for the measurement was sodium borohydride and dilute nitric acid. Nitric acid (65% mass fraction) and water were mixed in a proportion of 1 to 9 parts by volume.

Pb content: The concentration of lead in the samples was measured according to the Hungarian MSZ EN 15510:2017 standard [85] using an iCAP 7400 ICP-OES analyser (Thermo Fisher Scientific Inc. Waltham, MA, USA) and multi-element standard solutions at concentrations of 0, 5, 10, 100, 500 and 1000 ppb at a wavelength of 220.353 nm, which is typically used for lead measurements.

Macro and microelements

The measurements were carried out according to the Hungarian MSZ EN 15510:2017 standard [85], which is applicable for the determination of the elemental content of feed. Sample preparation was performed by dry ashing the samples and then measuring them using ICP-OES. A 5 g sample, weighed with 1 milligram accuracy, was transferred into a cremation container, which was placed in an incinerator at 450 °C for 3–4 h, and a white or grey ash was formed. The ash was washed with 30 mL of dilute hydrochloric acid in a 250 mL beaker and 100 mL of water was added. It was covered with a lid and cooked on a hob for 30 min. After cooling, it was washed into a 500 mL measuring flask and then the flask was filled with distilled water. The micro- and macroelement contents were determined by ICP-OES in the corresponding wavelength ranges: manganese: 257.610 nm; iron: 259.940 nm; copper: 324.754 nm; zinc: 213.856 nm; sodium: 589.592 nm; calcium: 315.887 nm; magnesium: 285.213 nm; and potassium: 766.490 nm. Standard solutions were prepared from a multi-element stock solution diluted with dilute hydrochloric acid at concentrations of 5, 10, 50, 100, 500, 1000 and 5000 ppb (1:1 cc. HCl and distilled water). The elements were analysed in two stages. Firstly, zinc, copper, iron and manganese, and then calcium, sodium, magnesium and potassium were analysed on the Y-branch with an ionisation aid (0.9% caesium and strontium chloride solution).

Statistical analysis

The IBM SPSS Statistics 26.0 (IBM Corp. Chicago, IL, USA) statistical software package was used to process and evaluate the data. The GLM (univariate) model was used to compare the means of the different measured values between treatments. The prerequisites for the dependent variables for analysis of variance were checked. The homogeneity of variance was tested using Levene’s test. LSD post hoc tests were conducted for the pairwise comparisons of the means. The significance level (alpha) was set to *p* = 0.05.

## 5. Conclusions

Watercress can be used in a variety of ways as a complementary component of intensive aquaculture systems. Our research has clearly demonstrated that in aquaponic environments contaminated with mercury or lead, watercress can accumulate significant amounts of heavy metals in its tissues. On the one hand, this raises health risk issues, as the contents of lead or mercury in the plant may be above the food safety standards. However, this shows that watercress is suitable for phytoremediation purposes because it can absorb large amounts of heavy metals from the water and significantly reduce the ecological risk of the run-off water used in aquaponic systems. This feature also enables it to be used as a natural filter in recirculation systems. The addition of humic acid to the system significantly altered its functioning and the element uptake of the watercress. Adding humic acid to the water used in aquaponics can reduce food safety risks, as the water tower will absorb significantly less mercury or lead from contaminated water in the presence of humic acid. Therefore, it would be useful to carry out process-level research on the stress factors affecting aquatic plants and their mitigation potential with humic acid or even other substances that do not negatively affect aquatic biota.

## Figures and Tables

**Table 1:** Sodium, potassium, manganese, copper and zinc contents of watercress shoots under mercury and humic acid treatments (Debrecen, 2023).

Element	Control	Hg	Hg + Humic Acid
Na (mg kg^−1^)	730.23 a	499.02 a	475.56 a
K (mg kg^−1^)	1109.79 a	838.84 b	1114.22 a
Mn (µg kg^−1^)	4209.09 b	2657.73 c	6423.92 a
Cu (µg kg^−1^)	3767.02 a	2486.87 a	2747.92 a
Zn (µg kg^−1^)	5363.51 b	4869.32 b	13,984.50 a

Different letters indicate statistically different values in the rows (*p* < 5%).

**Table 2:** Sodium, potassium, manganese, copper and zinc contents of watercress under lead and humic acid treatments (Debrecen, 2023).

Element	Control	Pb	Pb + Humic Acid
Na (mg kg^−1^)	730.23 a	494.13 b	524.77 b
K (mg kg^−1^)	1109.79 a	1129.36 a	1172.42 a
Mn (µg kg^−1^)	4209.09 b	3375.10 b	6454.31 a
Cu (µg kg^−1^)	3767.02 a	3281.22 a	2211.38 a
Zn (µg kg^−1^)	5363.51 a	5609.34 a	5287.56 a

Different letters indicate statistically different values in the rows (*p* < 5%).

## Data Availability

The data presented in this study are available on request from the corresponding author. The data are not publicly available due to the data are part of an ongoing study.
